# The Functional and Imaging Implications of Left Bundle Branch Pacing in Ischemic Cardiomyopathy

**DOI:** 10.3390/biom15040489

**Published:** 2025-03-26

**Authors:** Fulvio Cacciapuoti, Ciro Mauro, Ilaria Caso, Salvatore Crispo, Rossella Gottilla, Valentina Capone, Saverio Ambrosino, Ciro Pirozzi, Orlando Munciguerra, Mario Volpicelli

**Affiliations:** 1 Division of Cardiology, “A. Cardarelli” Hospital, 80131 Naples, Italy; 2 Department of Cardiology, “V. Monaldi” Hospital, 80131 Naples, Italy; 3 Department of Cardiology, “Santa Maria della Pietà” Hospital, 80035 Naples, Italy

**Keywords:** bundle branch pacing (BBP), heart failure with reduced ejection fraction (HFrEF), ischemic cardiomyopathy, cardiac resyncronization therapy (CRT)

## Abstract

Heart failure with reduced ejection fraction due to ischemic cardiomyopathy remains a significant clinical challenge. Electrical conduction delays exacerbate symptoms by causing uncoordinated contractions, reducing pumping efficiency, and increasing mortality. Right ventricular pacing further worsens dyssynchrony, while resynchronization therapy improves outcomes but has a high non-responder rate. Given these limitations, bundle branch pacing engages the heart’s conduction system, restoring synchronized contraction and enhancing cardiac function. This review examines the impact of left-bundle-branch-block-induced dyssynchrony, the role of advanced imaging in assessing ventricular function, and the clinical outcomes of bundle branch pacing in heart failure patients. Specifically, we explore the mechanical and hemodynamic effects of left bundle branch block, imaging techniques for dyssynchrony evaluation, and the comparative benefits of bundle branch pacing versus resynchronization therapy. Conduction delays impair function, increase myocardial stress, and worsen clinical outcomes. Advanced imaging plays a critical role in patient selection, identifying those most likely to benefit from conduction system pacing. By restoring electrical coordination, bundle branch pacing enhances ventricular function, reduces hospitalizations, and promotes reverse remodeling. It offers similar or superior benefits to conventional resynchronization therapy, regulates stress hormones, reduces oxidative damage, and improves calcium handling. Bundle branch pacing represents a significant advancement in heart failure management, but careful patient selection remains crucial. Future research should focus on optimizing implantation techniques and validating long-term benefits through large-scale clinical trials.

## 1. Introduction

Heart failure with reduced ejection fraction (HFrEF) secondary to ischemic cardiomyopathy represents one of the most significant challenges in modern cardiology. This condition contributes to a high number of hospitalizations and is associated with substantial morbidity and mortality, making it a critical target for therapeutic advancements [1]. Patients with concomitant left bundle branch block (LBBB) present an additional layer of complexity, as the disruption in ventricular synchronization leads to mechanical inefficiency and progressive remodeling [2]. This exacerbates heart failure symptoms, reduces exercise capacity, and increases overall mortality risk.

In LBBB, the delayed activation of the left ventricle results in mechanical dyssynchrony, causing inefficient contraction patterns [3]. The interventricular septum and lateral wall contract out of sync, leading to paradoxical septal motion that increases ventricular wall stress and accelerates adverse remodeling [4]. While traditional pacing techniques, such as right ventricular (RV) pacing, address bradycardia, they can often worsen dyssynchrony and further impair cardiac function. Similarly, cardiac resynchronization therapy (CRT) has demonstrated success in correcting ventricular desynchronization, yet its effectiveness is limited by a considerable non-responder rate and the complexity of implantation [5].

**Figure 1:**
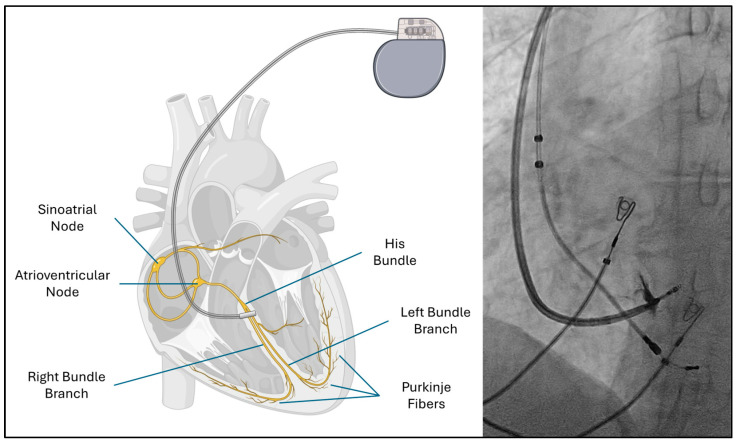
A schematic diagram of the cardiac conduction system with placement of left bundle pacing lead (**left**). Fluoroscopic image (**right**).

Bundle branch pacing (BBP) has emerged as a physiological alternative that directly engages the native conduction system through His bundle pacing or left bundle branch area pacing [6] (Figure 1). By restoring synchronized ventricular activation, BBP improves mechanical function and systemic hemodynamics. Early clinical evidence suggests this technique not only reduces dyssynchrony but also promotes reverse remodeling, decreases hospitalizations, and enhances the quality of life for patients with HFrEF [7].

## 2. Pathophysiology of Dyssynchrony in Left Bundle Branch Block

LBBB-induced dyssynchrony involves a complex interplay of electrical, mechanical, hemodynamic, and metabolic disturbances. The loss of synchronized ventricular activation leads to inefficient contraction, impaired relaxation, and structural remodeling, all of which worsen heart failure symptoms and increase arrhythmic risk [8]. Under normal conditions, the His-Purkinje system ensures rapid and simultaneous activation of both ventricles, allowing for coordinated contraction of the interventricular septum and lateral walls, which optimizes cardiac output. However, in LBBB, conduction through the left bundle branch is delayed or blocked, leading to a sequence of detrimental mechanical and hemodynamic changes [9].

**Figure 2:**
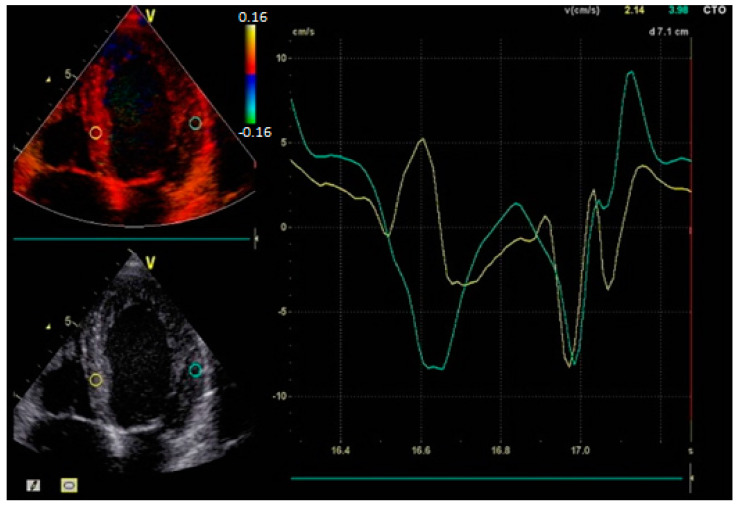
Echocardiographic assessment of a patient with left bundle branch block. Tissue Doppler imaging highlighting interventricular dyssynchrony.

As the right ventricle is activated normally via the right bundle branch, the left ventricle experiences a significant delay in depolarization. This forces electrical impulses to propagate through myocardial tissue rather than the fast-conducting Purkinje fibers, creating interventricular dyssynchrony where the ventricles contract at different times. Within the left ventricle, further desynchronization occurs as the septum and lateral wall contract out of phase. The result is inefficient contraction patterns characterized by early septal activation followed by late lateral wall contraction [10] (Figure 2).

**Figure 3:**
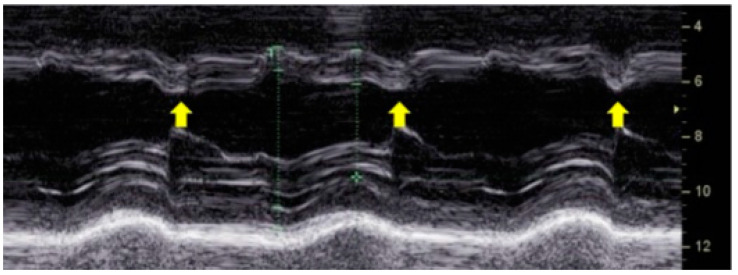
M-mode echocardiography showing septal flash, a characteristic sign of left bundle branch block. The yellow arrows indicate the early systolic septal motion abnormality caused by dyssynchronous ventricular activation.

A hallmark feature of LBBB-induced dyssynchrony is paradoxical septal motion. Early contraction of the right ventricle pulls the septum toward the left ventricle, while the delayed lateral wall contraction stretches the septum back toward the right ventricle. This phenomenon, known as “septal flash”, is a key echocardiographic finding associated with inefficient myocardial contraction [11] (Figure 3). The resulting uncoordinated motion increases myocardial wall stress and reduces left ventricular ejection efficiency, leading to progressive systolic dysfunction.

The mechanical inefficiencies extend beyond septal motion abnormalities. Delayed contraction of the lateral wall prolongs systole, impairing diastolic relaxation and reducing ventricular filling time, ultimately compromising stroke volume and cardiac output [12].

**Figure 4:**
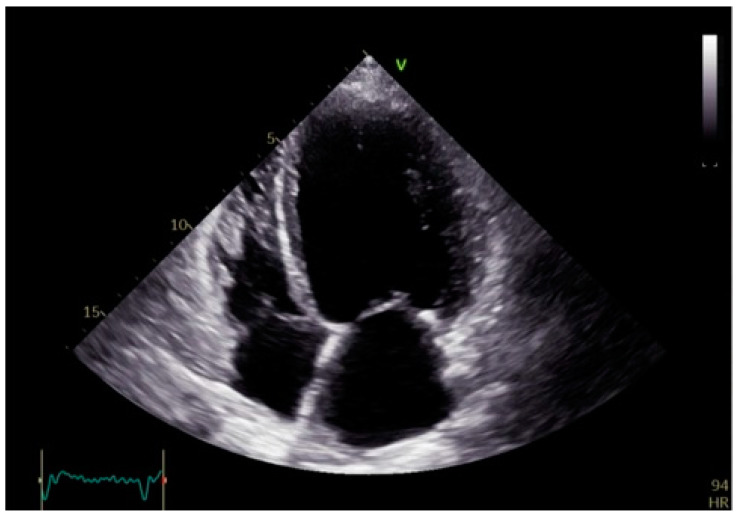
Apical four-chamber echocardiographic view showing dilated left ventricle with a fibrotic interventricular septum, characterized by increased echogenicity and reduced mobility, suggestive of structural remodeling.

Over time, the increased workload and wall stress lead to adverse remodeling, including left ventricular dilation, hypertrophy, and fibrosis (Figure 4). These structural changes further deteriorate heart failure symptoms, heighten arrhythmic risk, and contribute to poor clinical outcomes [13].

The hemodynamic burden imposed by dyssynchrony in LBBB is substantial. The loss of coordinated contraction reduces ventricular pump efficiency, leading to impaired systemic perfusion. Consequently, patients develop elevated left ventricular end-diastolic pressures, which contribute to pulmonary congestion and secondary right ventricular dysfunction. This perpetuates a cycle of worsening heart failure, limiting exercise capacity and increasing hospitalization rates.

Beyond mechanical and hemodynamic effects, LBBB-induced dyssynchrony also disrupts myocardial metabolism and perfusion [14]. The inefficient contraction pattern leads to suboptimal myocardial energy utilization, increasing oxygen demand while delivering less effective systolic function. Additionally, prolonged systole encroaches on diastolic time, disrupting coronary perfusion and exacerbating ischemia, particularly in patients with underlying coronary artery disease.

Clinical studies indicate that patients with LBBB experience a more rapid decline in left ventricular function compared to those with normal conduction. Moreover, LBBB is associated with an increased risk of sudden cardiac death as its dyssynchronous activation pattern creates a substrate for malignant ventricular arrhythmias [15]. All these factors emphasize the need for effective interventions to restore electrical synchrony in patients with heart failure.

Advancements in imaging techniques such as Tissue Doppler Imaging (TDI), speckle-tracking echocardiography (STE), and global longitudinal strain (GLS) have improved the ability to quantify dyssynchrony, enabling better patient selection for resynchronization therapies.

## 3. Imaging Modalities for Assessing Dyssynchrony

**Figure 5:**
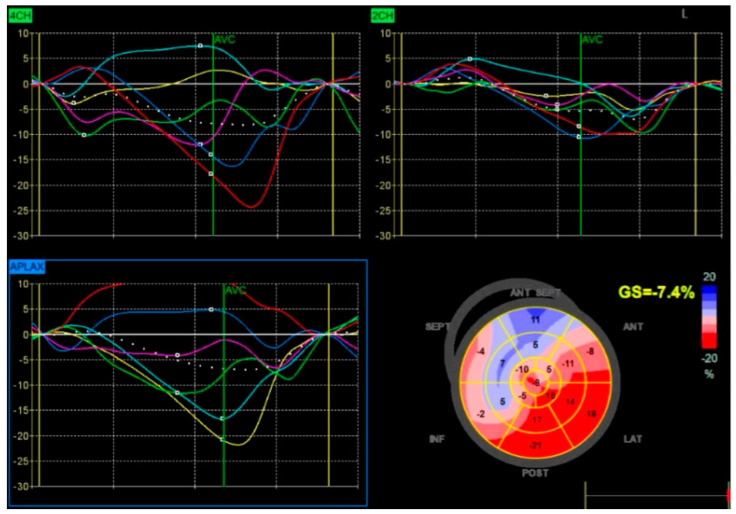
Speckle-tracking echocardiography showing global longitudinal strain analysis. The strain curves indicate significant dyssynchrony, and the bullseye plot reveals severely impaired GLS, suggesting advanced myocardial dysfunction.

Although a QRS duration of ≥130 milliseconds on electrocardiography is a key criterion for BBP suitability [16], echocardiographic advancements have greatly enhanced the assessment of ventricular mechanics and their response to treatment in patients with LBBB and HFrEF. Imaging plays a pivotal role in guiding patient selection for BBP or CRT, predicting treatment response, and monitoring the long-term success of resynchronization therapy [17]. The ability to detect subtle changes in myocardial mechanics, such as GLS improvements or reductions in septal-to-lateral delay, allows for individualized therapy optimization, ultimately improving patient outcomes [18] (Figure 5).

Tissue Doppler Imaging: TDI is a widely used modality for evaluating dyssynchrony by measuring myocardial velocities at different ventricular segments. This technique assesses the time-to-peak systolic velocity between the interventricular septum and the left ventricular (LV) lateral wall, providing an objective measure of intraventricular delay. In LBBB, this delay frequently exceeds 65 milliseconds, highlighting significant desynchronization [19].

A key finding in TDI is paradoxical septal motion, where early septal stretching is followed by delayed contraction due to premature right ventricular activation. This inefficient movement pattern is a hallmark of LBBB and contributes to mechanical dysfunction. TDI also enables a more comprehensive evaluation of dyssynchrony across other myocardial regions, such as the anterior and posterior walls, and serves as a valuable tool for monitoring improvements after CRT or BBP.

Speckle-Tracking Echocardiography: STE provides a refined evaluation of myocardial deformation by analyzing strain and strain rate. Unlike TDI, which measures myocardial velocities, STE evaluates the percentage change in myocardial length during contraction, offering a more precise assessment of ventricular mechanics [20].

In LBBB, GLS is often significantly reduced due to asynchronous myocardial contraction. STE generates strain maps illustrating areas of delayed or paradoxical motion. The interventricular septum typically exhibits reduced or negative strain in early systole, while the lateral wall demonstrates delayed peak strain, underscoring the extent of dyssynchrony [21]. This quantitative analysis aids in determining a patient’s likelihood of responding to resynchronization therapy.

STE has several advantages over TDI, including angle independence and greater accuracy in detecting subclinical LV dysfunction. Improvements in GLS following BBP or CRT are strongly correlated with better clinical outcomes, including enhanced LVEF, reduced ventricular volumes, and improved functional capacity [22].

**Figure 6:**
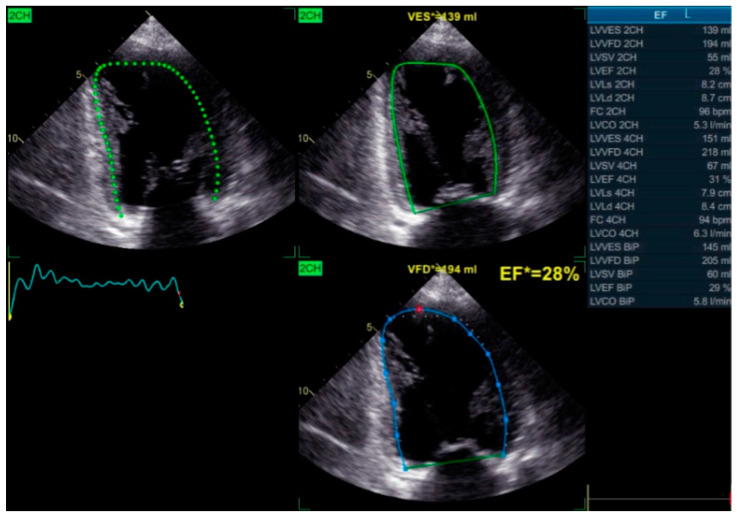
Biplane Simpson’s method echocardiographic assessment showing a reduced ejection fraction, indicating severe left ventricular systolic dysfunction.

Simpson’s Method for Ejection Fraction Assessment: The biplane method of discs, or Simpson’s method, remains a cornerstone for evaluating global LV function, particularly left ventricular ejection fraction (LVEF). This technique calculates LVEF based on end-diastolic and end-systolic volumes obtained from apical two- and four-chamber views (Figure 6).

In patients with LBBB, LVEF is frequently reduced due to inefficient contraction patterns. While Simpson’s method is less sensitive than TDI or STE in detecting regional dyssynchrony, it remains essential for assessing overall ventricular performance. Declining LVEF reflects adverse remodeling, whereas improvements following BBP or CRT indicate successful resynchronization and enhanced cardiac function.

Tricuspid Annular Plane Systolic Excursion (TAPSE): TAPSE is an essential echocardiographic measure of RV function, assessing the longitudinal movement of the tricuspid annulus during systole. In patients with LBBB and heart failure, TAPSE is often reduced due to increased RV workload caused by elevated pulmonary pressures and impaired ventricular interaction.

LBBB-related dyssynchrony indirectly affects RV function by increasing LV filling pressures, leading to secondary pulmonary hypertension and further deterioration of RV contractility. A reduction in TAPSE suggests worsening RV performance, while improvements post-BBP or CRT indicate better ventricular coupling, reduced pulmonary congestion, and enhanced hemodynamics.

**Figure 7:**
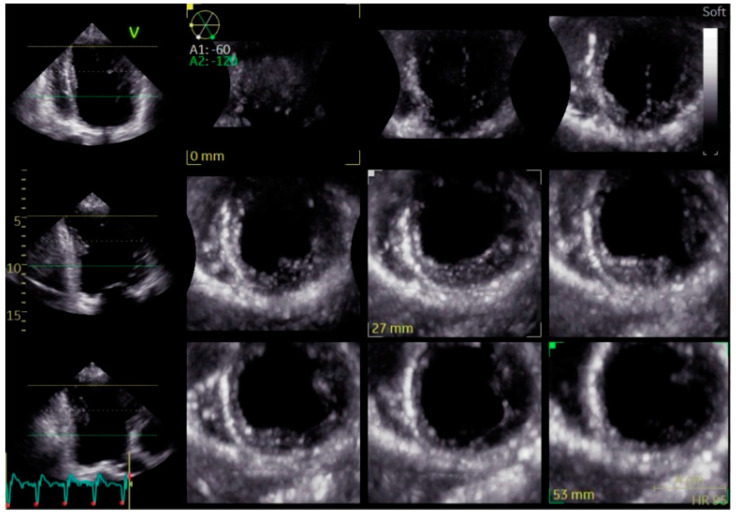
Three-dimensional, multi-slice transthoracic echocardiography showing short-axis views of the left ventricle at different levels. The images highlight increased echogenicity of the interventricular septum, suggestive of septal fibrosis, which may indicate myocardial remodeling.

**Figure 8:**
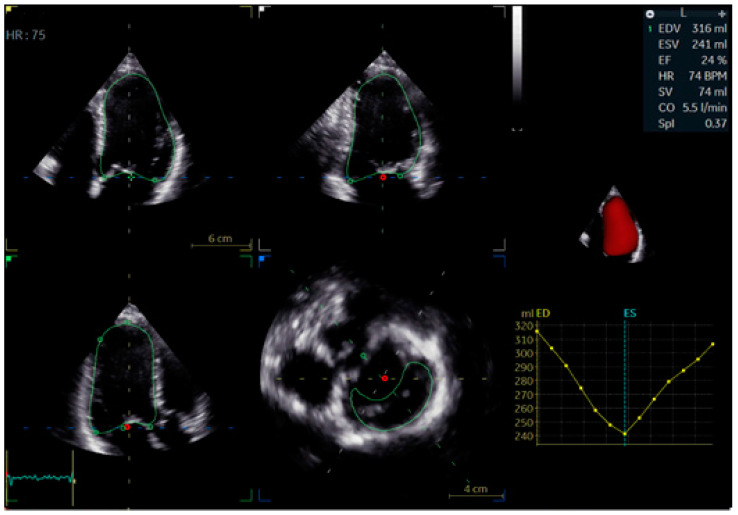
Three-dimensional, multi-view echocardiographic study in a patient with post-ischemic HFrEF.

Three-Dimensional Echocardiography (3D Echo): Three-dimensional echocardiography provides an advanced volumetric analysis of ventricular mechanics, offering a more comprehensive evaluation of dyssynchrony than conventional two-dimensional methods (Figure 7). Unlike standard echocardiography, 3D imaging captures time-to-peak contraction across multiple myocardial segments, allowing for a more detailed assessment of intraventricular coordination (Figure 8).

In LBBB, 3D echocardiography reveals the extent of mechanical dyssynchrony and its impact on ventricular geometry and volumes. This modality is particularly useful for tracking the effects of BBP or CRT as it can detect subtle changes in ventricular function and remodeling over time [23]. While its availability and expertise requirements currently limit widespread use, 3D Echo is expected to play an increasing role in dyssynchrony assessment and resynchronization therapy optimization.

Cardiac Magnetic Resonance Imaging (CMR): CMR remains the gold standard for assessing myocardial structure, ventricular volumes, and fibrosis. While echocardiography is the primary modality for dyssynchrony assessment, CMR provides superior accuracy in quantifying ventricular mass, identifying regional wall abnormalities, and detecting myocardial fibrosis [24]. Late gadolinium enhancement (LGE) imaging is particularly useful for identifying areas of fibrosis, which may impact the effectiveness of BBP or CRT [25]. Patients with extensive myocardial scarring are less likely to benefit from conduction system pacing, as fibrotic tissue disrupts the transmission of pacing impulses.

Recently, CMR has evolved to include advanced techniques such as 4D Flow MRI and CMR-derived strain imaging, both of which provide deeper insights into cardiac function and hemodynamics [26].

Four-dimensional Flow MRI enables comprehensive analysis of intraventricular blood flow dynamics, allowing for the assessment of energy dissipation, vortex formation, and mechanical efficiency. By visualizing and quantifying these parameters, 4D Flow MRI provides valuable information about ventricular workload, which is particularly useful in conditions like heart failure, valvular disease, and cardiomyopathies [27]. This technique helps refine our understanding of cardiac efficiency beyond traditional volumetric assessments.

CMR-derived strain imaging, on the other hand, offers a quantitative measure of myocardial deformation, similar to STE, but with higher spatial resolution and superior tissue characterization. It allows for precise detection of subtle myocardial dysfunction in diseases such as hypertrophic cardiomyopathy, myocarditis, and heart failure with preserved ejection fraction [28]. Unlike STE, CMR strain imaging is not limited by acoustic window quality, making it a more reliable tool in assessing global and regional myocardial mechanics.

Together, these advanced CMR techniques enhance the evaluation of cardiac performance, ventricular workload, and myocardial contractility, contributing to more refined risk stratification and personalized treatment strategies.

## 4. Clinical Outcomes of Bundle Branch Pacing

By restoring native conduction, BBP synchronizes ventricular activation, leading to significant improvements in symptom control, functional capacity, hospitalization rates, and reverse remodeling [29]. Furthermore, BBP provides systemic benefits, enhancing quality of life and long-term survival in patients with HFrEF and LBBB [30].

Patients with HFrEF and LBBB often experience dyspnea, fatigue, and exercise intolerance, which severely limit daily activities. The restoration of synchronized ventricular contraction via BBP enhances cardiac efficiency and reduces myocardial wall stress, alleviating heart failure symptoms.

One of the most widely documented benefits of BBP is improvement in New York Heart Association (NYHA) functional class. Many patients experience an upgrade of at least one NYHA class post-implantation, reflecting a clinically meaningful reduction in symptom burden and greater exercise tolerance [31]. The six-minute walking test also demonstrates notable increases in walking distance and reduced dyspnea following implantation. These gains translate into improved overall well-being as patients regain the ability to perform daily activities with less fatigue [32].

Heart failure decompensation leading to hospitalizations is a major burden for both patients and healthcare systems. Recurrent admissions are associated with progressive cardiac decline, worsening comorbidities, and increased mortality risk. BBP has been shown to significantly lower hospitalization rates by stabilizing heart failure symptoms, improving hemodynamics, and preventing disease progression [16]. Studies report up to a 50% reduction in heart failure-related hospitalizations following BBP implantation, underscoring its potential to decrease healthcare costs and improve long-term outcomes [33].

**Figure 9:**
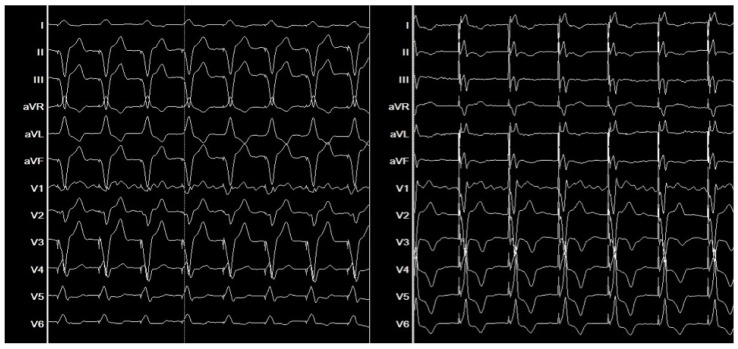
Electrocardiographic comparison showing left bundle branch block pattern before (**left**) and after (**right**) conduction system pacing. The post-pacing ECG demonstrates improved ventricular activation with narrower QRS complexes.

Unlike conventional RV pacing, which can exacerbate adverse remodeling [34], BBP preserves the integrity of the native conduction system, maintaining physiological ventricular activation patterns. In LBBB, prolonged QRS duration is a marker of dyssynchrony and inefficient contraction. By engaging the His-Purkinje system, BBP narrows QRS duration, synchronizing ventricular activation and enhancing myocardial performance [35] (Figure 9).

Electrocardiographic improvements post-BBP are not merely cosmetic but reflect fundamental enhancements in cardiac electrophysiology and mechanical function. Studies have demonstrated a strong correlation between QRS narrowing and improved hemodynamics, further supporting BBP as an effective resynchronization strategy [36]. However, in cases where conduction system disease is extensive or BBP fails to achieve appropriate capture, CRT offers an alternative approach by directly stimulating the myocardium, which can be particularly beneficial in patients with significant fibrosis or compromised conduction pathways (Table 1). Post-implantation electrocardiographic parameters provide valuable insights into pacing efficacy, with QRS narrowing serving as a key indicator of improved synchrony.

Additionally, reductions in ventricular activation time suggest more efficient propagation of electrical impulses, while repolarization changes can reflect modifications in myocardial electrophysiology, potentially influencing arrhythmic risk and overall cardiac function.

Beyond cardiac improvements, BBP positively influences other organ systems, particularly the kidneys and pulmonary circulation.

HFrEF patients frequently develop renal impairment due to chronic hypoperfusion and congestion. BBP enhances cardiac output, improving renal perfusion and resulting in lower creatinine levels and increased eGFR [37].

BNP and NT-proBNP, markers of myocardial wall stress and fluid overload, significantly decrease post-BBP, indicating improved hemodynamics and reduced congestion [38].

Patients often report better mental clarity, reduced fatigue, and enhanced energy levels, reflecting improved systemic perfusion.

All these benefits translate into increased long-term survival for patients with HFrEF and LBBB [39].

Although further research is needed to establish definitive long-term survival benefits, current evidence indicates BBP provides comparable or superior outcomes to CRT in selected patients, positioning it as a cornerstone therapy for conduction disturbances in appropriately selected patients, especially in those who are non-responders to CRT or have specific anatomic or electrical substrates favoring conduction system pacing.

This includes improvements in left ventricular function, reverse remodeling, symptom relief, and reductions in heart failure hospitalizations (Table 2).

## 5. Echocardiographic Selection Criteria and Evidence of Reverse Remodeling Following Bundle Branch Pacing

While BBP is an effective and physiological pacing strategy for many patients with HFrEF and LBBB, not all individuals are suitable candidates. A thorough echocardiographic evaluation is essential to identify anatomical, structural, or functional limitations that could reduce procedural feasibility, limit clinical efficacy, or increase the risk of complications [40]. Recognizing these challenges ensures that alternative pacing strategies, such as CRT or biventricular pacing, are considered in cases where BBP may not provide optimal benefits.

A major limitation to BBP candidacy is the extent of left ventricular fibrosis, particularly in patients with ischemic cardiomyopathy or advanced non-ischemic cardiomyopathies. When myocardial scarring disrupts the conduction system, the pacing stimulus may fail to propagate efficiently, making it difficult to achieve stable ventricular synchronization [41]. CMR imaging with LGE is the gold standard for detecting fibrosis, but echocardiography can also provide useful clues, including severe wall motion abnormalities, interventricular septal thinning below 6 mm, and reduced myocardial strain on STE [42]. A lack of contractile reserve on stress echocardiography further suggests that the affected myocardium is unable to respond effectively to pacing. In patients with transmural fibrosis, particularly in the septal or basal regions, conduction system capture may be unreliable or ineffective, making CRT a more appropriate alternative as it bypasses diseased pathways and provides broader electrical activation [43].

Another factor limiting the success of BBP is severe left ventricular dilation, commonly observed in advanced heart failure. When the left ventricular end-diastolic diameter exceeds 70 mm or the left ventricular end-systolic diameter is greater than 60 mm, extensive remodeling may prevent the pacing stimulus from effectively reaching the left bundle branch, reducing the likelihood of achieving synchronized ventricular activation. In such cases, biventricular pacing through CRT may be a more reliable alternative as it directly stimulates both ventricles and compensates for structural and mechanical alterations present in advanced heart failure.

The interventricular septum plays a crucial role in BBP lead fixation and conduction system engagement, and abnormalities in this region can interfere with pacing effectiveness. Patients with marked septal thinning, akinesia, or paradoxical septal motion without a contractile reserve may struggle to achieve stable pacing outcomes [44]. Additionally, the presence of extensive septal fibrosis detected on speckle-tracking or stress echocardiography may indicate that the conduction system is too damaged for BBP to provide meaningful hemodynamic improvements.

Right ventricular dysfunction and pulmonary hypertension also present challenges for BBP candidacy as these conditions often indicate broader biventricular failure, which may limit the benefits of left-sided resynchronization alone. Echocardiographic parameters such as TAPSE below 10 mm, right ventricular fractional area change under 20%, moderate-to-severe tricuspid regurgitation, and pulmonary artery systolic pressures exceeding 50 mmHg suggest that right ventricular impairment may reduce the overall hemodynamic benefits of BBP [45]. In such cases, CRT is often preferred because it supports both ventricles and improves biventricular function more comprehensively.

Elderly patients, especially those with severe diastolic dysfunction and restrictive filling patterns, may also experience limited symptomatic relief from BBP, as their left ventricles operate at persistently elevated filling pressures despite restoring electrical synchrony [46]. Mitral inflow Doppler patterns showing an E/A ratio greater than 2, an elevated E/e’ ratio above 14, or severe left atrial enlargement exceeding 50 mm indicate advanced diastolic dysfunction, which may not significantly improve with BBP alone. In such cases, CRT may offer greater symptomatic relief, particularly when atrial remodeling or restrictive physiology is a predominant factor in the patient’s heart failure progression.

Anatomy and pacing lead positioning are also critical considerations for BBP feasibility, as structural challenges can complicate implantation and reduce procedural success [47]. Prominent moderator bands or excessive septal trabeculation can interfere with lead fixation, making it difficult to achieve stable conduction system capture. Similarly, atrial septal patches or prosthetic valves can create physical barriers that prevent optimal lead placement [48]. In these situations, alternative pacing approaches, such as CRT or biventricular pacing, may provide more consistent and reliable resynchronization. A comprehensive echocardiographic evaluation ultimately allows for optimized patient selection, ensuring that each intervention is tailored to the patient’s specific cardiac pathology, improving long-term outcomes and quality of life [49].

Despite these limitations, BBP has been shown to promote reverse remodeling in appropriately selected patients. Echocardiographic assessments following BBP implantation demonstrate marked reductions in left ventricular end-diastolic volume and left ventricular end-systolic volume, reflecting a shift away from pathological dilation and a return to more efficient ventricular mechanics [50]. This structural improvement alleviates the burden on the heart and supports better hemodynamic performance.

In addition to volumetric changes, myocardial strain analysis provides valuable insights into BBP’s role in reversing dyssynchrony. STE has shown that BBP significantly improves GLS by enhancing the coordination of myocardial contraction. This improvement is closely linked to the restoration of electrical synchrony, aligning the contraction of the septum and lateral wall. Notably, GLS has been identified as a strong predictor of clinical outcomes in heart failure, with improvements following BBP correlating with better functional capacity, fewer symptoms, and increased survival [51].

Post-implantation echocardiography frequently demonstrates synchronous septal and lateral wall contraction, leading to more effective systolic function. This correction is particularly evident in TDI, which reveals resolution of early systolic stretching and delayed contraction in the septum. The normalization of wall motion reduces myocardial stress and enhances overall pump efficiency, which is essential for long-term cardiac recovery.

Another key structural benefit of BBP is the reversal of increased ventricular sphericity, another hallmark of progressive remodeling in HFrEF. As the LV dilates, its shape changes from an ellipsoid to a more spherical configuration, increasing wall stress and further impairing contraction. BBP has been shown to restore a more favorable ventricular geometry, reducing wall tension and improving contractile mechanics [52]. This shift supports better overall LV function and contributes to the long-term benefits of BBP in heart failure management.

## 6. Biomolecular Effects of Bundle Branch Stimulation

Beyond the well-documented mechanical and electrical benefits, BBP induces a series of biomolecular changes that affect myocardial remodeling, cellular metabolism, and electrical stability [53].

One of the most significant effects of BBP is its ability to modulate fibrotic remodeling [54]. In ischemic HFrEF, the loss of cardiomyocytes due to ischemic events and persistent neurohormonal activation lead to excessive collagen deposition in the extracellular matrix, resulting in increased left ventricular stiffness and progressive deterioration of contractile function [55]. This fibrotic process is primarily mediated by the overexpression of transforming growth factor-beta (TGF-β1) and the activation of cardiac fibroblasts [56]. By promoting a more coordinated contraction, bundle branch stimulation reduces mechanical stress disparities on the ventricular walls, limiting TGF-β1 activation and modulating the expression of matrix metalloproteinases (MMPs) and their inhibitors (TIMPs) [57]. This mechanism slows down fibrotic progression and helps preserve better left ventricular compliance.

Another crucial aspect is the improvement in intracellular calcium homeostasis. In patients with ischemic HFrEF and LBBB, myocardial calcium release is disorganized due to asynchronous left ventricular activation [58]. This disruption compromises contractility and relaxation, contributing to diastolic dysfunction. BBP restores a more physiological activation sequence, enhancing the synchronization of calcium release from the sarcoplasmic reticulum through ryanodine receptor 2 (RyR2) and increasing the activity of sarcoplasmic reticulum Ca^2+^-ATPase (SERCA2a) [59]. This process improves calcium reuptake, leading to enhanced myocardial contraction and relaxation efficiency.

Simultaneously, BBP induces metabolic adaptations in ischemic myocardium. The electrical–mechanical decoupling in LBBB increases energy consumption, which, under chronic ischemic conditions, exacerbates the metabolic deficit in cardiomyocytes [60]. CRT and BBP reduce this energy expenditure by improving contraction coordination and optimizing subendocardial perfusion [61]. Additionally, studies have demonstrated that resynchronization can enhance mitochondrial function, reducing oxidative stress and limiting cellular damage from free radicals [62].

Another major impact of bundle branch stimulation is its modulation of neurohormonal pathways, which are severely dysregulated in ischemic HFrEF. Chronic activation of the sympathetic nervous system and the renin–angiotensin–aldosterone system contributes to pathological cardiac remodeling, increasing left ventricular workload and promoting heart failure progression [63]. BBP mitigates sympathetic overactivation, reducing circulating norepinephrine levels and enhancing parasympathetic tone. These effects not only improve cardiac function but also decrease the risk of arrhythmic events, which are often linked to excessive sympathetic activity [64].

Finally, a critical aspect of BBP is its influence on electrical stability in ischemic myocardium. Ischemic LBBB is associated with altered expression of connexins (Cx43, Cx45), the proteins responsible for electrical conduction between cardiomyocytes [65]. This disorganization increases repolarization heterogeneity, predisposing patients to ventricular arrhythmias [66]. Both CRT and BBP restore connexin expression and distribution, improving electrical signal transmission and reducing the risk of ventricular tachycardia and fibrillation [67].

## 7. Challenges and Future Directions

Despite its promising benefits, BBP faces challenges related to procedural complexity and patient selection. Implantation requires precise mapping and lead placement, which can be technically demanding. Variability in response among patients underscores the need for improved selection criteria, particularly in identifying individuals with extensive myocardial scarring. Future research should focus on refining implantation techniques, standardizing protocols, and conducting large-scale trials to establish the long-term benefits and safety of BBP. Advances in imaging technology and mapping tools may further enhance the precision and success of this innovative approach.

BBP is a transformative innovation in the management of HFrEF and LBBB. The effects of BBP go beyond merely correcting electrical activity detectable on ECG. BBP significantly improves left ventricular performance by enhancing synchrony and reducing mechanical inefficiencies. Additionally, its biomolecular effects play a crucial role in slowing the progression of HFrEF by modulating myocardial remodeling, optimizing calcium handling, and reducing oxidative stress.

In combination with novel pharmacological agents such as vericiguat, BBP may also contribute to the preservation of connexin expression and distribution—particularly connexin-43—which is essential for maintaining efficient electrical coupling between cardiomyocytes. This combined approach may enhance myocardial conduction, reduce arrhythmogenic risk, and ultimately improve systolic function through both mechanical and molecular pathways. However, this area remains insufficiently explored, and further research is needed to better understand the interplay between conduction system pacing, pharmacologic modulation, and gap junction biology in heart failure.

For these reasons, BBP could represent a paradigm shift in the management of conduction disturbances in heart failure, offering a more physiological and effective alternative to conventional pacing strategies. With continued advancements in imaging, device technology, and patient selection criteria, BBP is poised to become a cornerstone therapy in the management of HFrEF with LBBB, ultimately improving outcomes for this high-risk patient population. While BBP offers promising advantages over CRT in selected patients, its widespread adoption requires further validation through long-term clinical trials assessing survival benefits and arrhythmic risk.

## Figures and Tables

**Table 1:** Key electrophysiological effects of BBP vs. CRT.

Electrophysiological Effect	Bundle Branch Pacing (BBP)	Cardiac Resynchronization Therapy (CRT)
Conduction System Engagement	Utilizes the His-Purkinje system, preserving conduction integrity	Direct myocardial stimulation, bypassing conduction system
Ventricular Activation Pattern	More physiological, mimicking natural ventricular depolarization	Non-physiological but enhances synchrony in dyssynchronous ventricles
QRS Duration	Narrower QRS compared to traditional pacing	QRS narrowing possible but may remain wider than BBP
Ventricular Activation Time	Reduced due to activation through native pathways	Can reduce activation time but depends on lead placement
Repolarization Effects	Minimal repolarization abnormalities	Potentially alters repolarization due to non-specific myocardial activation
Effect in Extensive Fibrosis	Limited effectiveness if conduction system is severely diseased	More effective in cases with significant fibrosis or conduction block

**Table 2:** Comparative outcomes of BBP vs. CRT: electrophysiological and clinical benefits.

Clinical Outcome	Bundle Branch Pacing (BBP)	Cardiac Resynchronization Therapy (CRT)
Changes in QRS Duration	Significant QRS narrowing by engaging the conduction system	QRS narrowing depends on lead placement; may not be as physiological as BBP
LVEF Improvements	Moderate improvement, particularly in patients with conduction disease	Greater improvement, especially in patients with LBBB and wide QRS
NYHA Class Improvements	Mild to moderate improvement in symptoms	More substantial symptomatic improvement, particularly in NYHA class III-IV
Reduction in Heart Failure Hospitalizations	Reduces hospitalizations in select patients, but less studied than CRT	Well-documented reduction in heart failure hospitalizations
Reverse Remodeling	Modest reductions in LV volumes due to improved contraction efficiency	More pronounced reductions in LVEDV and LVESV due to improved synchrony
Arrhythmia Burden	Lower risk of ventricular arrhythmias; uncertain effect on atrial fibrillation	May reduce ventricular arrhythmias but can increase atrial fibrillation risk

## Data Availability

The datasets are available from the corresponding author on reasonable request.

## Abbreviations

The following abbreviations are used in this manuscript:

BBP	Bundle Branch Pacing
HFrEF	Heart Failure with Reduced Ejection Fraction
TDI	Tissue Doppler Imaging
STE	Speckle-Tracking Imaging
GLS	Global Longitudinal Strain
CRT	Cardiac Resynchronization Therapy
LBB	Left Bundle Branch Block
